# Successfully managed bilateral pneumothorax and subcutaneous emphysema after emergency open surgical tracheotomy

**DOI:** 10.1186/s42077-021-00136-8

**Published:** 2021-02-19

**Authors:** Fulya Yilmaz

**Affiliations:** University of Health Sciences Izmir Bozyaka Training and Research Hospital, Department of Anesthesiology and Reanimation, Saim Çıkrıkçı Caddesi, No. 59 35170 Karabağlar, İzmir, Turkey

**Keywords:** Bilateral pneumothorax, Subcutaneous emphysema, Emergency open surgical tracheostomy

## Abstract

**Background:**

The most common complications we encountered in tracheotomies are hemorrhage, pneumothorax, and tube displacement. In this case report, we describe bilateral pneumothorax following an emergency tracheotomy.

**Case presentation:**

A 57-year-old woman, who was diagnosed with laryngeal carcinoma, was developed sudden respiratory distress in the ear nose throat (ENT) ward before surgery. The patient was taken to the operating room for emergency tracheotomy. After surgery, at the 5th minute of the mechanical ventilator follow-up in ICU, she developed subcutaneous emphysema on her eyes, face, neck, and chest. She was taken to re-operation. On the postoperative follow-up, bilateral pneumothorax was detected on chest X-ray and bilateral thorax tube was applied by thoracic surgeon. She was externed to ENT ward on the 3rd postoperative day. The left thorax tube was removed on the 2nd and right thorax tube was removed on the 6th postoperative day.

**Conclusion:**

Here, we presented a successfully managed bilateral pneumothorax and subcutaneous emphysema after emergency open surgical tracheotomy. If persistence reduction of SPO_2_ levels after tracheotomy, pneumothorax should be kept in the mind.

## Background

The most common complications we encountered in tracheotomies are hemorrhage, pneumothorax, and tube displacement. Pneumothorax may develop especially in children and in patients with chronic lung diseases (Cipriano et al. 2015; Kasugai et al. 2016). The risk of complications in emergency tracheotomies increases 2 to 5 folds (Sreelakshmi 2018). In this case report, we describe bilateral pneumothorax following an emergency open surgical tracheotomy (EOST).

## Case presentation

**Fig. 1:**
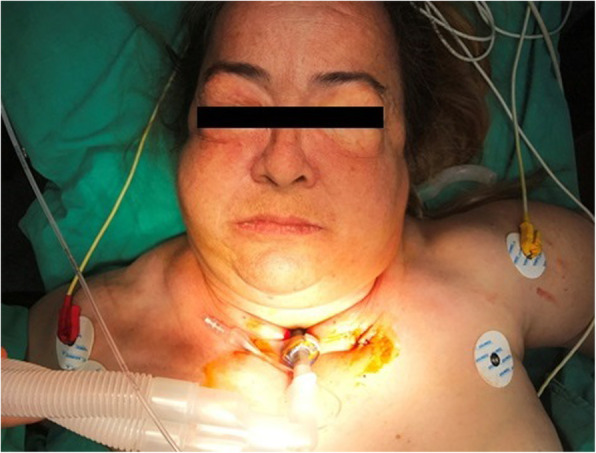
Acute subcutaneous emphysema

**Fig. 2:**
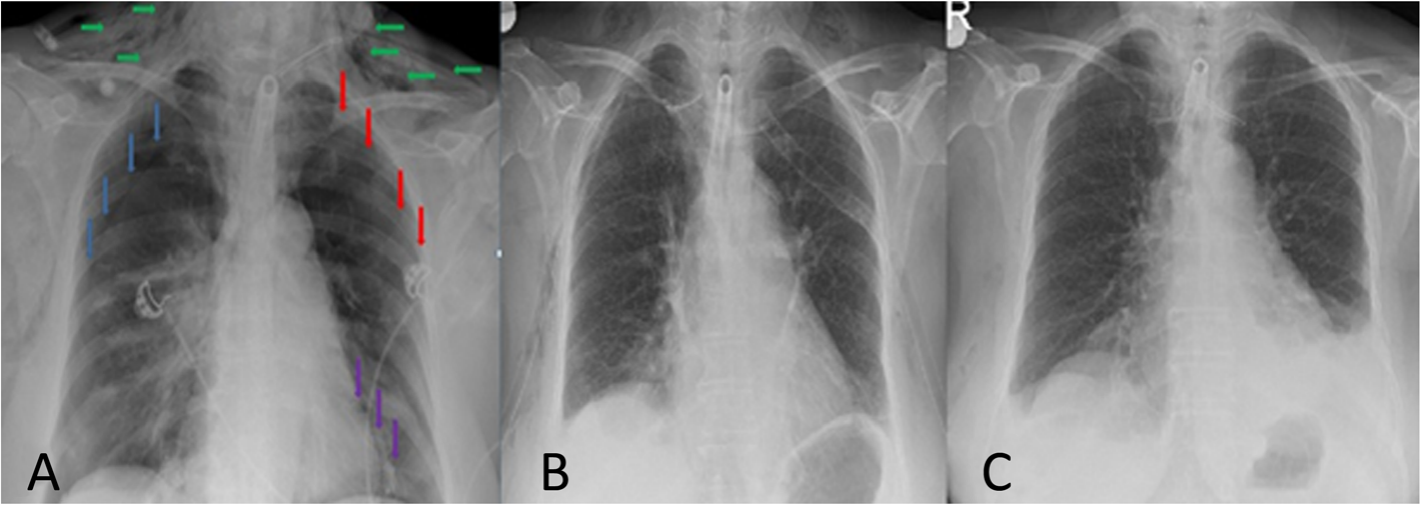
Bilateral pneumothorax, bilateral thorax tube application, and after removal of throax tubes X-Ray. **a** Bilateral pneumothorax (red and blue arrows) and subcutaneous emphysema (green arrow), **b** bilateral thorax tube application, and **c** chest X-ray after removal of throax tubes

Written informed consent was obtained from the patient for hid anonymized information to be published in this case report. A 57-year-old woman (weight 85 kg, height 157 cm), who was diagnosed with laryngeal carcinoma, developed sudden respiratory distress in the ENT ward before surgery. Besides history of smoking, she had no other medical or surgical history. The patient was transported to the operation room for EOST under spontaneous ventilation with 100% oxygen supplement with mask ventilation support. The patient’s blood pressure was 165/80 mmHg, and heart rate was 95 beats min^−1^ and SpO_2_ was 85% when she was monitored in the operation room. We did not attempt to intubate the patient because the ENT doctor declared that it is impossible to intubate the patient because of the mass effect. So we applied urgent tracheotomy under sedoanalgesia. 7.0 tracheotomy cannula was placed by the ENT doctor in the day shift with difficulty because of short neck, obesity, and mass of laryngeal carcinoma under sedoanalgesia (propofol boluses of 20 mg intravenously (total 140 mg) and remifentanil 0.1 μg kg^−1^ min^−1^ infusion) and local anesthesia with 15 mL of prilocaine. We also administered methylprednisolone (100 mg) and ranitidine hydrochloride (50 mg) intravenously. After the tracheotomy cannula placed, we checked ventilation by auscultation in the operation room and we applied rocuronium (0.5 mg/kg) intravenously before transporting the patient to the intensive care unit (ICU) for postoperative follow-up on mechanical ventilation. The patient’s blood pressure was 135/75 mmHg, and heart rate was 75 beats min^−1^ and SpO_2_ was 95% after the procedure. Mechanical ventilation was set to adaptive support ventilation (ASV) in the ICU (the patient weight, height, and gender were entered; PEEP, 5 mmHg; FiO_2_, 50%; percentage of the minimum volume of 100%) under dexmedetomidine infusion. At the 5th minute of the mechanical ventilator follow-up, she developed subcutaneous emphysema on her eyes, face, neck, and chest (Fig. 1). We suspected cannula displacement. She was taken to the operation room again with respiratory difficulty, cyanosis, and low saturation (SpO_2_ < 30%) under mask ventilation with 100% oxygen supplementation. We transported the patient to the operation room again because the initial tracheotomy application was difficult and our ICU was near the operation room. ENT doctor applied 7.0 tracheal cannula again but it was not inserted to the trachea because of the long depth of the neck. They inserted a 6.5 spiral endotracheal tube for trachea. Then, anesthesia was maintained by remifentanil 0.2 μg kg^−1^ infusion with 6% desflurane in 55% oxygen and air after 100 mg propofol bolus. Rocuronium boluses were repeated to facilitate the surgery. A tracheal stoma was created in an hour and 6.5 spiral endotracheal tube was replaced with an 8 cuffed tracheal cannula. After the operation, she was taken to the ICU for postoperative follow-up on mechanical ventilation (adaptive support ventilation; PEEP, 7 mmHg; FiO_2_, 60%) again under sedoanalgesia and muscle relaxation. She continued low saturation (SpO_2_ < 90%) levels on the first hour of the postoperative follow-up. Immediate chest X-ray was planned. Bilateral pneumothorax was detected on chest X-ray and bilateral thorax tube was applied by thoracic surgeon (Fig. 2). She was discharged to ENT ward on the 3rd postoperative day. The left thorax tube was removed on the 2nd and right thorax tube was removed on the 6th postoperative day.

## Discussion

Negative pressure in upper airway obstructions, intermittent positive pressure ventilation (IPPV) after tracheotomy, direct pleural injury, tube displacement, or other etiology causing mediastinal air leakage are the causes of bilateral pneumothorax following tracheotomy (Cipriano et al. 2015; Kasugai et al. 2016; Jain et al. 2014). We thought direct pleural injury (related with procedure or cannula displacement), cannula displacement itself, or intermittent positive pressure ventilation were probable causes in our case.

In a case report, the authors detected hemi-lateral pneumothotrax, pneumomediastinum, and subcutaneous emphysema by chest X-ray and CT on the second day of emergency tracheotomy. They managed the patient successfully (Takasugi et al. 2018).

But in another case report, authors reported a fatal case of tension pneumothorax (a large right-sided pneumothorax) and subcutaneous emphysema after open surgical tracheostomy (Gupta and Modrykamien 2014).

In conclusion, here, we presented a successfully managed bilateral pneumothorax and subcutaneous emphysema after emergency open surgical tracheotomy. If there is a persistent reduction of SPO_2_ levels after tracheotomy, pneumothorax should be kept in the mind.

## Data Availability

The datasets used and/or analyzed during the current study are available from the corresponding author on reasonable request.

## Abbreviations

EOST: Emergency open surgical tracheotomy
ENT: Ear nose throat
